# Renal Endometriosis: The Case of an Endometrial Implant Mimicking a Renal Mass

**DOI:** 10.1089/cren.2018.0070

**Published:** 2018-10-29

**Authors:** Anand V. Badri, Rachel Jennings, Pavan Patel, Daniel D. Eun

**Affiliations:** ^1^ Department of Urology, Temple University Hospital, Philadelphia, Pennsylvania.; ^2^ Lewis Katz School of Medicine, Temple University, Philadelphia, Pennsylvania.; ^3^ Cooper Medical School of Rowan University, Camden, New Jersey.

**Keywords:** renal endometriosis, partial nephrectomy, gross hematuria

## Abstract

***Background:*** Endometriosis is a multifactorial benign disorder characterized by the abnormal presence of endometrial tissue in an extraendometrial site. Although extrapelvic endometriosis is uncommon, symptomatic involvement of the kidney is exceedingly rare. This benign disease can mimic several urologic processes, but because of its scarcity in clinical practice, it is seldom considered in the differential.

***Case Presentation:*** In this report, we describe the case of a 45-year-old woman with flank pain and hematuria, who was found to have a left renal mass on cross-sectional imaging. After robotic partial nephrectomy, pathologic analysis revealed an endometrial implant within the renal parenchyma.

***Conclusion:*** This case of renal endometriosis highlights how this benign disease process can mimic several more sinister urologic processes.

## Introduction

Endometriosis, the abnormal growth of endometrial tissue in an extraendometrial site, is a common entity, although not readily seen in most urologic practices; it represents the most common cause of chronic pelvic pain in women and the second most common pelvic pathology in women.^1^ Endometriosis, an estrogen-dependent process, classically manifests during reproductive years with cyclical symptoms of dysmenorrhea, dyspareunia, and dyschezia correlating with menstruation.^2^ Cases of extrapelvic endometriosis have been reported and in this study we present an exceedingly rare case of renal endometriosis.^1^

## Case Presentation

**FIG. 1.**
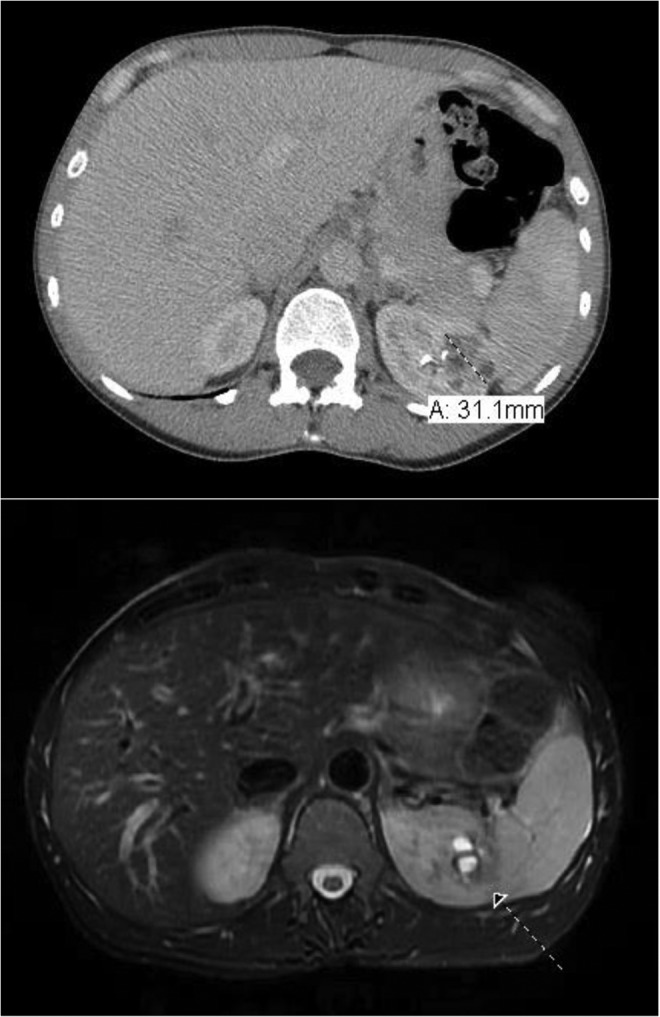
(*Top*) Axial CT image capturing the 3.1 cm *left upper pole* renal mass. (*Bottom*) Axial MRI T2 sequence demonstrating cystic components to the renal lesion. *Arrow* indicates the renal mass.

In August 2017, a 45-year-old woman presented to our outpatient urology clinic in consultation for intermittent gross hematuria associated with flank pain. Her medical history was significant for antiphospholipid syndrome and her surgical history was notable for previous tubal ligation; the remainder of her history was unremarkable. She had not undergone menopause. No abnormalities were noted on abdominal and pelvic physical examination. Laboratory work-up revealed a hemoglobin of 12.0 (g/dL) and an estimated glomerular filtration rate >60 (mL/min/1.73 m^2^). Cystoscopic evaluation was unremarkable. To complete her hematuria evaluation, a triphasic CT was obtained demonstrating a 3.1 cm left upper pole heterogeneous, partially enhancing renal mass (R.E.N.A.L Nephrometry Score = 9X). Subsequent abdominal MRI was performed reaffirming the presence of the renal mass and demonstrating cystic components with parenchymal enhancement (Fig. 1). The differential diagnoses derived from the radiologic findings at this time included a renal malignancy, a benign renal mass, multilocular cystic nephroma, or sequelae from a prior focal pyelonephritis.

We reviewed diagnostic and treatment options, most notably active surveillance, renal biopsy, ablative therapy, and partial nephrectomy. Given the cystic nature of the lesion, along with her young age, we recommended robotic partial nephrectomy.

The patient was placed into a modified flank position and using a standard left-sided robotic kidney port placement, the left kidney was exposed, and the mass was readily identified. Intraoperative ultrasonography was used to delineate the echogenic renal mass and the single renal artery was clamped before sharp excision of the lesion. A visually appreciated negative margin was maintained throughout the resection of the mass. The mass was noted to be cystic and loculated with the deep margin penetrating toward the collecting system, requiring a larger rim of resection than normally anticipated for a 3 cm renal mass. After renorrhaphy, the mass was then extracted and sent for pathologic determination.

Pathology revealed a 2.8 × 2.6 × 1.7 cm partially cystic lesion that was encapsulated with negative margins. Immunostains for desmin, CD10, Melan-A, and HMB-45 were performed confirming a diagnosis of endometriosis with smooth muscle metaplasia.

## Discussion

Although extrapelvic endometriosis is uncommon, symptomatic involvement of the urinary tract is rare, and few cases have been reported in the literature.^3^ Case data suggest the urologic organ distribution to be bladder (80%), ureter (15%), and kidney/urethra (<5%)^3^ with most of these cases affecting women aged 25–40 years.^1^

Histopathologic changes in endometriosis can aid in diagnosis. Ectopic endometrial tissue thickens and sheds in response to hormonal changes in the menstrual cycle, and when manifested in a renal implant, may mimic intrinsic renal pathologies. Cyclical shedding in any location of the urinary tract could lead to gross or microscopic hematuria. A fibrotic tissue rind is often formed with the additional accumulation of hemorrhagic cysts resulting in the formation of an endometrioma in the kidney; if the renal capsule becomes involved, flank pain may ensue.^2^ The endometrioma itself accounts for the imaging manifestations, which, when combined with a similar symptomatic course, largely accounts for its unexpected diagnosis, as seen with our patient.

In summary, renal endometriosis is a rare manifestation of a common disease that can mimic several urologic processes. The presentation of symptoms such as hematuria, renal colic, flank pain, and ureteral obstruction correlate with a large urologic differential, but because of its rarity in clinical practice, renal endometriosis is seldom considered in the differential. To further complicate the clinical picture, imaging features of renal endometriosis typically do not facilitate an accurate diagnosis; one cannot differentiate between endometriosis and a cystic malignancy on ultrasonography, CT, and MRI. That being said, when a patient presents with urologic symptoms, along with imaging that demonstrates a mass, the clinical picture can lead a clinician to favor extirpation given concern for malignancy. Renal endometriosis should be suspected when the symptoms reviewed are cyclical in nature, correlate with menses, and the patient is of appropriate reproductive age. If a patient presents with a high clinical suspicion for possible renal endometriosis, a percutaneous renal mass biopsy may be offered, obviating the need for invasive procedures.^4^ For symptomatic lesions, renal mass ablation or partial nephrectomy could be recommended. To avoid misdiagnosis, an awareness of endometriosis and its symptoms are essential to the clinician as diagnosis relies heavily on a high index of clinical suspicion.

In an asymptomatic patient, a renal mass biopsy followed by active surveillance may have been pursued as an appropriate form of management. However, in our case, given the patient's persistent symptoms, we effectively managed this patient with partial nephrectomy. Although a renal mass biopsy could have been a useful diagnostic option, we opted not to biopsy because of the cystic nature of our patient's renal mass. Postoperatively, the patient recovered without any complications and remains symptom-free on her last follow-up visit.

## Conclusion

We present an extremely rare and interesting case of a patient who presented with flank pain, hematuria, and a renal mass. This case of renal endometriosis highlights how this benign disease process can mimic several more sinister urologic processes. As the depth of literature for renal endometriosis is poor, we hope to increase the scholarly awareness of this entity to practitioners.

## Abbreviations Used

CT: computed tomography
MRI: magnetic resonance imaging
